# Cefoperazone rapidly and sensitive quantitative assessment via a validated RP-HPLC method for different dosage forms, in-use stability, and antimicrobial activities

**DOI:** 10.1186/s13065-023-00989-0

**Published:** 2023-07-12

**Authors:** Mostafa F. Al-Hakkani, Nourhan Ahmed, Alaa A. Abbas, Mohammad H. A. Hassan

**Affiliations:** 1 Department of Chemistry, Faculty of Science, Al-Azhar University, Assiut Branch, Assiut, 71524 Egypt; 2 Department of Research, Development, and Stability, UP Pharma, Industrial Zone, Arab El Awamer, Abnoub, 76, Assiut, Egypt; 3 Department of Medical Laboratory Technology, Higher Technological Institute for Applied Health Sciences in Minya, Minya, Egypt

**Keywords:** Antimicrobial, Cefoperazone, Detection, Quantitation, Stability

## Abstract

Cefoperazone (Cfz) is a member of the third generation of parenteral cephalosporin antibiotics. It is used on a wide scale in prescribed antibiotic drugs as anti-infection, especially for Gram-negative and also against Gram-positive microorganisms. The current study aimed to find a rapid RP-HPLC method of Cfz analysis with high linearity, repeatability, sensitivity, selectivity, and inexpensive. In our developed method, there is no need to use special chemical reagents, a high percentage of organic solvent, a high flow rate, further guard column. The chromatographic system comprises an ODS column (150 mm × 4.6 mm × 5 μm). The mobile phase was prepared by mixing KH_2_PO_4_ solution: acetonitrile (80:20) with a flow rate of 1.0 mL/min at detection wavelength 230 nm, at room temperature using injection volume 20 μL. The method manifested a satisfied linearity regression R^2^ (0.9993) with a good repeatability range (0.34–0.92%) with LOD and LOQ; 4.04 μg/mL and 12.24 μg/mL respectively. The method proved its efficiency via system suitability achievement in the robustness and ruggedness conduction according to the validation guidelines. The shorter analysis time makes the method very valuable in quality control to quantify the commercial Cfz in pharmaceutical preparations. This improved HPLC method has been successfully applied for Cfz analysis for Peracef and Peractam vials in our routine finished and stability studies testing laboratories. Additionally, the detection limit of Cfz has been tested in our quality control lab to detect the smallest amount of traces that may be present after the cleaning process of the production machines for cephalosporins preparations. In a precedent for the first time, we were able to use the current analysis method to determine the minimum inhibitory concentration (MIC) and minimum bacteriostatic concentration (MBC). The conventional broth micro-dilution tube method was used to determine MIC at 250 µg/mL and MBC at 125 µg/mL of Cfz against the standard strain of *Burkholderia cepacia (B. cepacia)* ATCC 25416 as Gram-negative bacteria in vitro.

**Supplementary Information:**

The online version contains supplementary material available at 10.1186/s13065-023-00989-0.

## Introduction

**Fig. 1:**
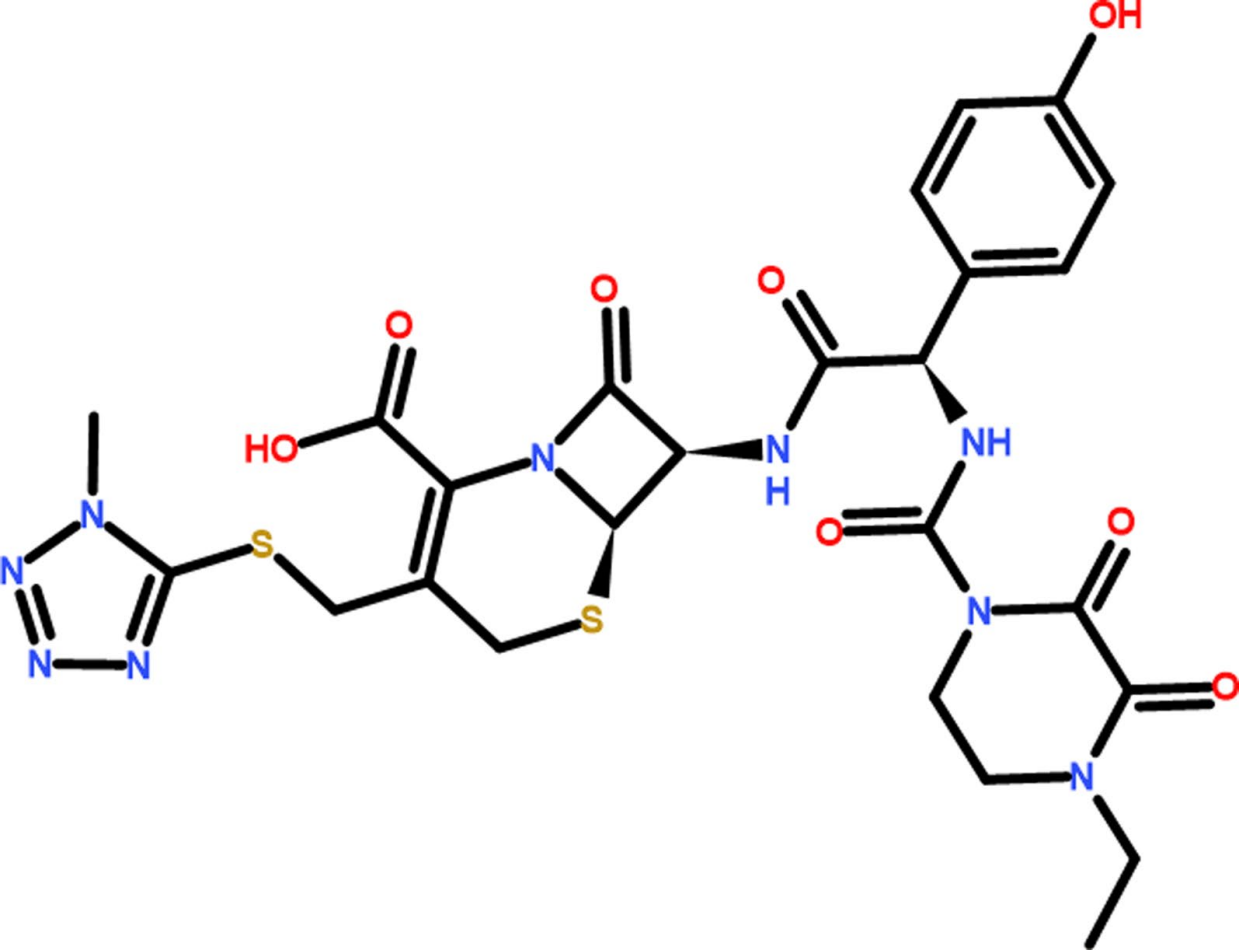
Structure of Cfz “C_25_H_27_N_9_O_8_S_2_ in molar mass “645.7 g/mole”

Cefoperazone(Cfz) is a semisynthetic, broad-spectrum antibiotic, and it has the IUPAC name [(6*R*,7*R*)-7-[[(2*R*)-2-[(4-ethyl-2,3-dioxopiperazine-1-carbonyl)amino]-2-(4-hydroxy-phenyl)acetyl]amino]-3-[(1-methyltetrazol-5-yl)sulfanylmethyl]-8-oxo-5-thia-1-azabi-cyclo[4.2.0]oct-2-ene-2-carboxylic acid]. It is the third-generation cephalosporin family [1] and it has the chemical molecular formula C_25_H_27_N_9_O_8_S_2_ in molar mass "645.7 g/mole" as manifested in Fig. 1.

Cfz could be taken by injection into a muscle, a vein, or an intravenous drip. Cfz is used in cases of infections of the respiratory tract, urinary tract, female reproductive system, and skin infections and it is known as an antibacterial, *especially Gram-negative, as it* is very effective in particular *Pseudomonas* and *Haemophilus influenza*. Where Cfz prevents the production of bacterial cell walls [2]. Also, it is used in Gram-positive infections such as *Staphylococcus aureus* and *Streptococcus pneumonia* [3]*.* Although the use of antibiotics is important in our lives, some antibiotics, especially cefoperazone, have an effective effect on some important enzymes in the metabolism as Glutathione S-transferase and acetylcholinesterase. As a result, utilizing this drug may have certain negative consequences for some people. Drug dosages need to be appropriately controlled to reduce negative effects. Patients who lack these enzymes due to a hereditary condition should be closely watched [4].

Cfz is sold under more than one brand name, such as Cefobid, Peracef, Cefazon, and Cefrazon with different strengths of 2.0 g, 1.0 g & 0.5 g vials. Cfz also can be found in mixed forms (cefoperazone sodium: sulbactam sodium) such as Trexotaz 2.0 g vials in the ratio (1:1) or as in Peractam, Sulperazon, Sulbacef as 1.5 g vials in a ratio (2:1).

The wide spectrum of this drug makes it important in the field of pharmaceutical trade, which necessitates the need to find effective, simple, easy, and rapid methods for assay determination. In addition, a sensitive method should be conducted at low concentrations of this drug preparation, when this method is used to estimate Cfz after washing cleaning machines and production lines. The sensitive method should be conducted to ensure the effectiveness of the cleaning method to remove the drug residual effects of this drug that may be entered into the next product in the production process, causing a completely unacceptable cross-contamination process [5–15]. This type of contamination is according to the quality standards mentioned in the rules of good manufacturing practice (GMP) [6, 7, 15].

There are many methods with more than one technique in the analysis tools being conducted for the assay determination of Cfz, including flow injection analysis with thermal lens spectrometry [16], HPTLC technique [17–20], UPLC-MS/MS [21], RP-HPLC [1–3, 22–25], silver nanoparticles spectrophotometrically [26], spectrophotometrically [27–29]. HPLC–MS [30].

However, HPLC–UV detection is an easy, accurate, and inexpensive method, both at an academic and commercial level rate. The United States-Pharmacopoeia (USP44-NF 39 2021) and British-Pharmacopoeia (BP-2022-VOL1) issued two different methods for determining Cfz. The USP44-NF39 2021 mobile phase is composed of triethylamine, glacial acetic acid, ACN, 1 N acetic acid, and water with a stationary phase column of 4.0-mm × 30 cm containing packing L1 at a flow rate of about 2 mL per minute. The retention time is about 22 min. On the other hand, the BP-2022-VOL 1 mobile phase consists of triethylammonium acetate, acetic acid, ACN, and water with a stationary phase column as 4.6-mm × 15-cm end-capped octadecylsilyl silica gel for chromatography R (5 µm) at flow rate about 1 mL per minute. The retention time is about 15 min.

Most of the Cfz HPLC conducted analysis methods used a high percentage of the organic modifiers from MeOH or ACN, adjusted pH buffer solutions, or special reagents such as tetrabutylammonium hydroxide. These factors are used to get the optimum peak shape with ideal tailing [19, 25].

The field of scientific research has recently tended to purify industrial wastewater, pharmaceutical factories, and hospitals, especially for antibiotics. So, finding easy, fast, accurate, and economical methods has become an urgent necessity [5–11, 15, 31–33].

In this paper, an efficient, simple, and rapid method for the assay determination of Cfz will be issued. Furthermore, inexpensive chemicals with integration analysis method parameters were used to obtain the detection and identification of Cfz. The current method became the approved analysis method to determine the Cfz in all the quality control lab activities that contain Cfz as starting raw material, finished product, and stability studies. Also, the method was successfully implemented in the cleaning validation and verification after the production of any pharmaceutical preparation containing Cfz. For the first time, we could determine the MIC and MBC using the present Cfz HPLC analysis method against the bacterial standard strain of *(B. cepacia).*

## Materials and methods

Cfz sodium working standard was supplied as a gift sample from UP Pharma (Assuit, Egypt). ACNHPLC-grade, Potassium dihydrogen phosphate, Hydrochloric acid 37%, Sodium hydroxide, and Hydrogen peroxide 30% (Scharlau, Spain). Water for injection (WFI) was used in the analysis and passed through a 0.45 μm nylon membrane filter before use. Phosphate solution was prepared by weighing about 5.82 g of potassium dihydrogen phosphate in 1000 mL of WFI.

### Chromatographic system configuration

Compared with the previously conducted HPLC methods and the current analysis method, we did not use a high percentage of the organic modifier of ACN, dedicated pH solution adjustment, or special chemical reagent to realize the optimum separation for the ideal system suitability achievement.

Cfz assay determination was conducted using the HPLC model HP 1100 series with variable wavelength. The current method was conducted with the RP C18 ODS column (150 mm × 4.6 mm × 5 μm) (Thermo Scientific). The mobile phase was prepared as "KH_2_PO_4_ solution "(5.82 g in 1000 mL of WFI)": ACN in a ratio (80:20, v/v) at a flow rate of 1.0 mL/min with detection wavelength at 230 nm at room temperature and injection volume 20 μL.

### Parameters of method validation

The HPLC validation method was performed according to the International Conference on Harmonization (ICH) guidelines concerning parameters including system suitability, Range of linearity, the limit of detection (LOD), the limit of quantification (LOQ), repeatability (precision), recovery and accuracy, robustness, ruggedness, the stability of the solution, specificity, and selectivity [12–14, 34, 35].

#### System suitability check

System Suitability was performed by injecting six replicate injections of the same sample solution which was prepared by dissolving a quantity of Cfz sodium equivalent to 1.0 g of Cfz [stock solution] in 1000 mL of WFI, then diluting 10 mL into 100 mL volumetric flask using of WFI to get a solution with concentration about 100 µg/mL.

#### Range and linearity

The analytical approach is deemed to be linear if there is a substantial portion between the response and claimed working concentration starting at the lowest point in the tested range and increasing to the highest point with R^2^ ≥ 0.999 [6, 7, 12–15, 34, 35].

Regression linearity equation:

1 $$\mathrm{Y }=\mathrm{ a X }\pm \mathrm{ b}$$

where Y represents the response of the average peak area, X represents the claimed working concentration in (%), a represents the slope and b is the intercept of the calibration curve.

The linearity parameter was submitted using five concentrations in the range (50–150%) of the Cfz working standard. The claimed working concentrations were prepared as, 50, 70, 100, 120, and 150 µg/mL using the WFI as a solvent from the stock solution at a concentration of 1000 µg/mL. Every solution was injected into duplicates.

#### Limit of detection (LOD)

It was defined as the lowest specific analyte concentration in the matrix that could be identified using the detection of the instrument. Furthermore, it should not be included in the accuracy, precision, and linearity ranges [12–14, 34–36].

#### Limit of quantitation (LOQ)

It was defined as the lowest specific analyte concentration in the matrix that could be identified using the detection of the instrument. Furthermore, it must be included in the accuracy, precision, and linearity ranges [12–14, 34–36].

LOD and LOQ could be calculated according to the slope and standard error data from the linearity of the calibration as the following:

2 $$\mathrm{LOD}=3.3\upsigma /\mathrm{S}$$

3 $$\mathrm{LOQ}=10\upsigma /\mathrm{S}$$

where σ: is the standard error of X & Y arrays and S: represents the slope of the linearity calibration curve.

#### Accuracy and recovery

Both recovery and accuracy are used alternatively. The measurement’s accuracy is defined as the proximity of the actual concentration (measured value) to the theoretical concentration (true value) [12–14, 34, 35].

Accuracy was implemented by weighing three individual Cfz working standards to give theoretical concentrations at (70 µg/mL), (100 µg/mL), and (120 µg/mL). Accuracy % could be estimated using the linearity equation:

4 $$\mathrm{Accuracy\%}=\mathrm{ Actual \,Conc}.\mathrm{\% }/\mathrm{ Theoretical \,Conc}.\mathrm{\% }\times 100$$

5 $$\mathrm{Actual\, Assay \%}=\mathrm{ Peak \,area \,of \,the \,test}/\mathrm{Mean\, peak \,area \,of \,the \,working} \times \mathrm{working \,weight}/\mathrm{test \,weight}\times 100$$

#### Repeatability and precision

Repeatability was conducted using six different preparations individually of the target concentration of the intended method (100% = 100 µg/mL) of using the same equipment on the same day via the same analyst or compared with another analyst as inter precision [12–14, 34, 35].

#### Robustness

Robustness was submitted using designed small changes including slight changes in the temperature, composition of the mobile phase, etc.

The designed small changes were conducted in a different organic solvent ratio (ACN) at (20% ± 2.5%) and KH_2_PO_4_ solution (80% ± 2.5%) and a flow rate (1 mL/min ± 0.1 mL/min).

#### Ruggedness

Ruggedness was submitted using designed and major observable changes including analyst-analyst, column-column, and day-day with maintaining all the analysis method parameters and conditions as it is without changes [37].

#### Stability of the mobile phase

This test was performed to judge the stability of the mobile phase composition over time. The test was conducted at the target concentration of (100% = 100 µg/mL) over 14 days. The mobile phase is valid to use at room temperature over 14 days the retention time, USP tailing, and theoretical plates within the acceptance criteria of the conducted experiments.

#### Specificity and selectivity

Forced degradation studies were performed to indicate the stability-indicating properties. Also, to ease verification and distinguishing the active substance under study “Cfz” from the possibility of its presence among many applied catalytic degradation products. Accelerated degradation was implemented using acid hydrolysis of 0.1 M HCl for 30 min, base hydrolysis of 0.1 M of NaOH for 30 min, accentuate oxidation degradation of 3.0% w/v of H_2_O_2_ for 30 min, and light degradation over 6 h.

### Test of the validated method

#### Cfz analysis of the different commercial dosage forms in the Egyptian local market

Peracef 1 g vials, Peractam 1.5 g vials, Trexotaz 1.5 g vials, and Sulbacef 1.5 g vials were tested using the validated method of Cfz.

#### Peracef 1.0 g vials batch number (220001) after the constitution and after dilution stability studies

The after-constitution stability study was conducted using the supplied solvent WFI at zero time, 24 h at room temperature 30 ± 2 °C, and in the refrigerator at temperature 5 ± 3 °C for 24 h. Also, the after-dilution stability study was conducted in the refrigerator at the same previous time interval using the solutions for infusion as shown in Table 1.

**Table 1:** The used solutions for after-constitution and after-dilution stability studies

Item	Manufacturer	Batch number	Concentration	Volume	Manufacturing date	Expiry date
WFI	Rameda	201516	–	5 mL	06/20	06/23
Sodium chloride	FIPCO	B32044#B	0.9 wt/v	500 mL	03/22	03/25
Glucose	Al Mottahedoon pharma	121C107	5% wt/v	500 mL	03/21	03/24
Glucose	FIPCO	D32002#A	10% wt/v	500 mL	05/22	05/24
Ringer	Al Mottahedoon pharma	2200276	–	500 mL	07/22	07/25

The constituted vial was performed using 5 mL of the WFI, then all the contents of the vial were transferred into a 1000 mL volumetric flask. Then a dilution of 20 mL of the constituted solution (1 mg/mL) in a 200 mL volumetric flask using WFI was conducted and introduced to the HPLC for assay in a final theoretical concentration (0.1 mg/mL of Cfz).

On the other hand, the after-dilution study was conducted individually for each solution as the following, all the vial content (theoretically 1000 mg of Cfz) in 500 mL of each pack. Then a dilution of 10 mL of the diluted Cfz solution (2 mg/mL) in a 200 mL volumetric flask was performed using WFI and introduced to the HPLC for assay in a final theoretical concentration (0.1 mg/mL of Cfz).

#### Determination of minimum inhibitory concentration (MIC) and minimum bacteriostatic concentration (MBC) of Cfz using HPLC-validated method

The conventional broth micro-dilution tube method was used to determine the minimum inhibitory concentration (MIC) and minimum bacteriostatic concentration (MBC) of Cfz against the standard strain of *Burkholderia cepacian (B. cepacian)* ATCC 25416 as Gram-negative bacteria in vitro [38].

An overnight culture of Gram-negative bacteria, *B. cepacian* ATCC 25416, was inoculated in Mueller–Hinton broth, adjusted to equivalent turbidity of 0.5 McFarland standards. Five different concentrations of Cfz (1000, 500, 250, 125, and 62.5 μg/mL) were diluted in five tubes (1 to 5) containing Muller–Hinton broth medium and used for testing their antimicrobial activities [38–41].

Under aseptic conditions, the prepared bacterial suspension was inoculated in the tested tube containing the different concentrations of the tested antibiotic. The tubes were incubated at 37 °C for 24 h.

The antimicrobial activity was assessed by visual observation of turbidity [42], moreover measuring the assay activity for the Cfz peak by the current validated HPLC method.

## Results and discussions

### System suitability check

**Fig. 2:**
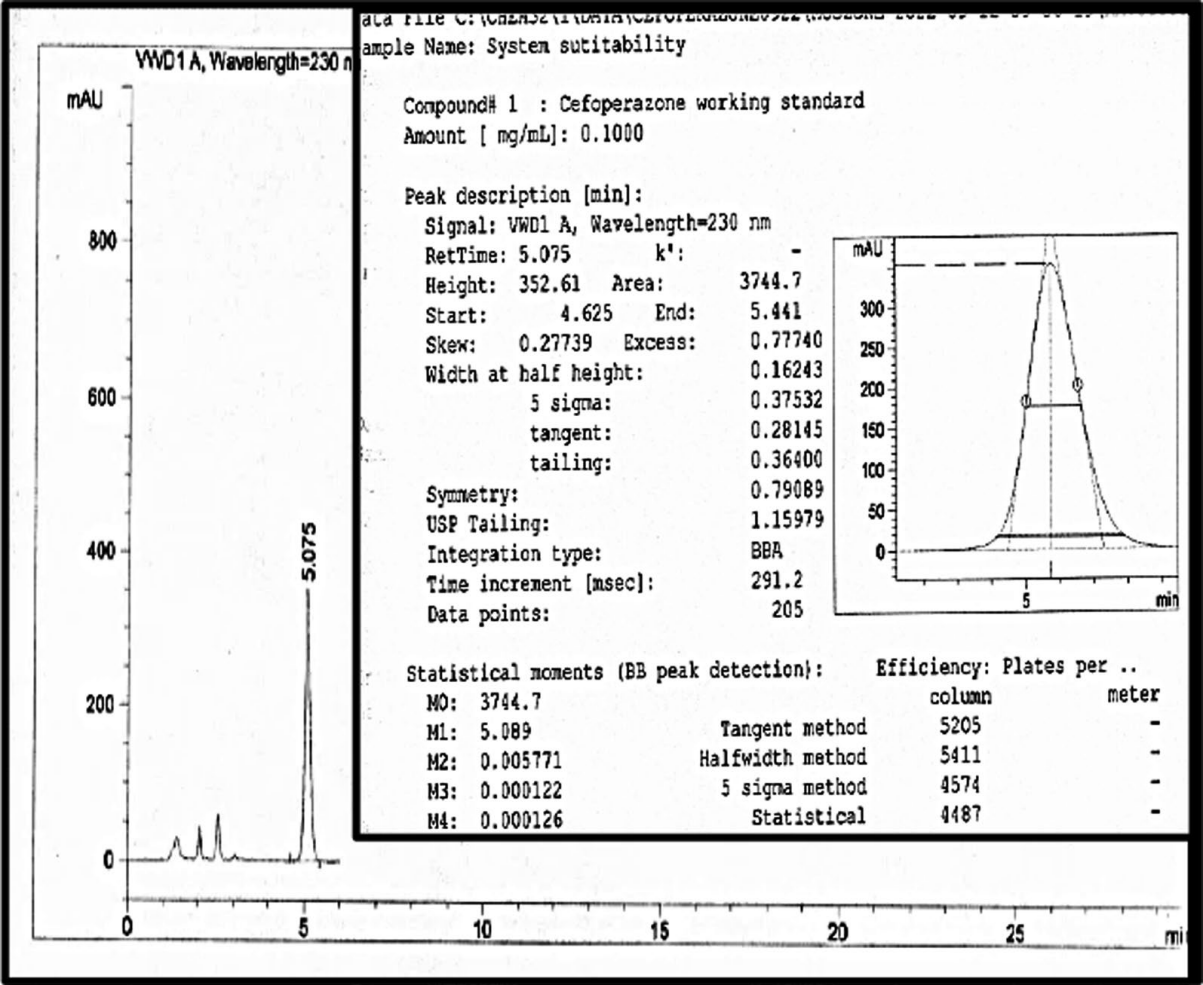
Cfz chromatogram at a flow rate 1 mL/min, Column—1, Buffer 80% & Acetonitrile 20%

Cfz peak appeared about at 5.0 ± 0.1 min at the optimum parameters of the analysis method Fig. 2 and in the range of (2.9–7.2) ± 0.1 min over all the parameter changes shown in Additional file 1: Fig. S1a–g. Table 2 showed high performance for the intended analysis method where the RSD% < 2.0%, USP tailing < 2.0, and theoretical plates ≥ 2000 [6, 7, 12–15, 34, 35]. So, according to the output data of the system suitability parameters, the method manifested superior validity through a wide range of retention times.

**Table 2:** System suitability

Replicate	Flow (1.0 mL) Buffer 80% First analyst Day—1 Column—1	Flow (0.9 mL)	Flow (1.1 mL)	Buffer (82.5%)	Buffer (77.5%)	Second analyst	Day—2	Column—2	Column—3
Weight(g)/100 mL	0.01094	0.01094	0.01094	0.01092	0.01094	0.01091	0.01095	0.01097	0.01091
1	3744.7	4138.2	3443	3779.1	3517.1	3734.7	3794.2	3864.8	3765.9
2	3746.1	4115.3	3441.3	3756.2	3486	3735.2	3782.9	3896.9	3781.2
3	3730.9	4164.9	3434.5	3784.0	3513.5	3741.6	3846.9	3908.0	3806.3
4	3785.2	4155.2	3444.2	3784.5	3515.4	3722.9	3794.1	3905.9	3799.9
5	3791.3	4128.8	3435.8	3754.8	3554.2	3723.5	3759.1	3887.8	3796.6
6	3753.8	4133.0	3429.5	3754.7	3524.5	3747.1	3737.3	3904.3	3859.8
Mean P.A	3758.7	4139.2	3438.1	3768.9	3518.5	3734.2	3785.8	3894.6	3801.6
STDV	24.2	18.1	5.7	15.1	21.9	9.6	37.3	16.4	32.0
RSD	0.64	0.44	0.17	0.40	0.62	0.26	0.98	0.42	0.84
Tailing	1.15979	1.20087	1.15984	1.14816	1.08537	1.11781	1.11781	1.08023	0.93333
Plates	5411	5700	4864	6078	4915	5179	5179	5135	5017

### Range and linearity

**Fig. 3:**
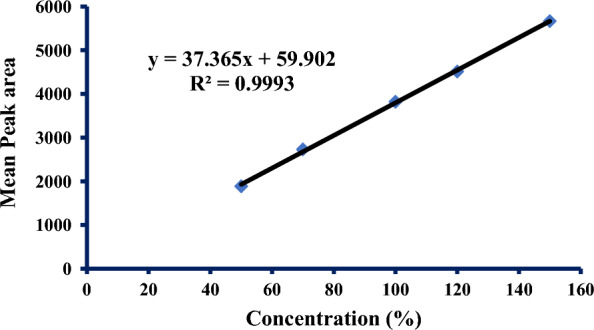
Linearity calibration curve of Cfz

The results manifested high linearity with R^2^ = 0.9993 at the working concentrations in the range (50–150%) as we can see in Fig. 3 and Table 3.

**Table 3:** Range and linearity

Working conc %	Average P. As	Statistical data	
50%	1884.4	R^2^	0.9993
70%	2728.0	Slope	37.365
100%	3819.5	Intercept	59.902
120%	4511.1	Standard error	45.726
150%	5665.5		

### LOD and LOQ

LOD and LOQ limits could be determined simply by using the linearity calibration data of Cfz. LOD was found to be 4.04 µg/mL whereas LOQ was 12.24 µg/mL.

### Accuracy and recovery

Table 4 showed that the accuracy results of the tested range (70–120% from the target concentration of 100% = 100 µg/mL) were found to be within the acceptance criteria (98–102%) [6, 7, 12–15, 34, 35].

**Table 4:** Accuracy and recovery

Theoretical Conc. %	Actual Peak area	Mean peak area	Theoretical wt (g)	Actual wt (g)/100 mL	Actual Conc. %	Accuracy %
70	2677.4	2683.0	0.00766	0.00767	70.29	100.4
	2692.5					
	2679.1					
100	3763.5	3757.9	0.01094	0.01097	99.24	99.2
	3758.4					
	3751.7					
120	4512.2	4517.0	0.01313	0.01318	119.74	99.8
	4520.8					
	4518.1					

### Repeatability and precision

The RSD% of peak areas was used for judgment on the repeatability of the analyte using six different preparations at the same target (100 µg/mL of Cfz) concentration as in Additional file 1: Table S1. It was found to be 0.39% and 0.34% within intra-precision and 0.92% at the inter-precision as it demanded in repeatability requirements RSD% < 2.0% [6, 7, 12–15, 34, 35].

### Robustness

The results of conscious small changes included a flow rate ± 0.1 mL/min and ACN (± 2.5%) was determined using RDS%. The RSD% was found to be < 2% in all cases as shown in Additional file 1: Tables S2 and S3. It is clear that is a reverse proportion between the retention time and the ratio of the organic modifier of the ACN. Where the retention time increases by decreasing the organic ratio and vice versa. This assures the principle chromatographic rule "likes to dissolve likes or likes attract likes"[12, 14, 34, 35].

### Ruggedness

The results of conscious major and observable changes include day-day and column-column. Data was presented as shown in Additional file 1: Tables S4 and S5. RSD% was found to be < 2% in all cases [12–14].

### Stability of mobile phase composition

The experimental results revealed that the tested solution of Cfz could is given repeatable and precise system suitability data over 14 days at room temperature as shown in Additional file 1: Table S6.

### Specificity and selectivity

The current method supplied us with highly specific data about the resolution and separation performance of the adjacent co-eluted peaks for the Cfz principle peak with a resolution parameter of at least 1.84 as shown in Additional file 1: Table S7 and Fig. S2a–2d.

Table 5 shows a comparison among some of the HPLC analysis methods procedures to determine Cfz. As we can see in our method there is no need to use of special chemical reagent, high organic percentage, pH adjustment of the mobile phase, post-column, pre-column, or high temperature. Additionally, the presented retention time is very acceptable with the time-saving analysis method.

**Table 5:** Comparison of HPLC procedures for the Cfz determination

Mobile phase Reagent	Organic, (ratio %)	Column	Flow rate (mL/min)	Detection (nm)	pH adjustment	Retention time (min)	Refs.
Potassium dihydrogen phosphate	ACN, 20	ODS—150 mm × 4.6 mm, 5.0 µm	1.0	230	Without	5.0 ± 0.1	Current
Tetraethylammonium acetate	MeOH, 35	Bonded β-CD (Cyclobond I), 250 mm × 4.6 mm, 5.0 μm + postcolumn	0.8	280	4.5	28.3	[2]
Tetramethylammonium hydroxide	MeOH, 25	ODS—120 mm × 4.0 mm, 2.5 µm	1.0	230	3.4	7.9	[3]
Phosphate buffer	MeOH, 55	C18—250 mm × 4.6 mm, 5.0 µm + Guard column	0.85	260	3.2	23.7	[22]
Phosphate buffer	ACN, 65	C8—150 mm × 4.6 mm, 5.0 μm	1.0	215	3.5	4.2	[23]
Tetrabutyl ammonium hydroxide	ACN, 25	C18—150 mm × 4.6 mm, 3.5 µm	1.0	230	6.8	20	[24]
Ammonium acetate	ACN, 5	C8—250 mm × 4.6 mm, 5.0 μm	0.8	250	5.6	23.58	[25]

### Test of the validated method

#### Cfz analysis of the different commercial dosage forms in the Egyptian local market

The Cfz assay results of Peracef 1 g vials, Peractam 1.5 g vials, Trexotaz 1.5 g vials, and Sulbacef 1.5 g vials revealed good results, as manifested in Additional file 1: Table S8. Where the method was found to be selective, specific, and resoluted for the Cfz peak either in the presence of sulbactam ingredients.

#### Peracef 1.0 g vials batch number (220001) after the constitution and after dilution stability studies

The tabulated results in Additional file 1: Tables S9–S13 confirmed the stability and validity of the use of the Cfz solutions either after constitution using WFI at 30 ± 2 °C and 5 ± 3 °C for 24 h or after dilution using different solutions such as sodium chloride 0.9% wt/v, Glucose 5% wt/v, Glucose 10% wt/v, and Ringer solutions. Where the assay% was found to be within the acceptance criteria and did not exceed 3.0% from the starting assay at zero time. Also, the results manifested that the method did not affect the composition of the different initiators of the solvent on the retention time over the study.

#### Observations of *B. cepacia*

The MIC test demonstrates the lowest level of an antibacterial agent that greatly inhibits growth; the MBC demonstrates the lowest level of an antibacterial agent resulting in microbial death [39–41].

After the MIC determination of the Cfz, aliquots of 50 μL from all the tubes which showed no visible bacterial growth were seeded on Tryptic soy agar plates and incubated at 37 °C for 24 h. When the bacterial growth is not observed at the lowest concentration of an antibacterial agent, it is termed an MBC endpoint. This was done by observing the inoculated plate's pre and after-being incubated for the presence or absence of bacterial growth.

After 24 h of incubation as it was manifested in Additional file 1: Fig. S3, no growth was observed in the negative control, turbidity was noticed in test tube no 5 containing a concentration of 62.5 μg/mL, and tube no 4 containing a concentration of 125 μg/mL of Cfz antibiotic, indicating the growth of bacteria. Whereas the tube from (1 to 3) contained conc of 1000, 500, and 250 μg/mL, no turbidity was seen, exhibiting inhibition of bacterial growth, meaning that the MIC was obtained at 250 μg/mL.

The suspension from the tubes of 250, 500, and 1000 μg/mL was inoculated on the Tryptic soy agar plate and incubated for 24 h. The result showed bacterial growth in the plate inoculated from tube no 3 containing the concentrations of 250 μg/mL, whereas no bacterial growth was observed in the other concentrations 500 and 1000 μg/mL, hence confirming it as bactericidal.

#### Determination of the MIC and MBC of Cfz using the HPLC-validated method

Fortunately, and according to our literature survey, we have reported the determination of the MIC and MBC of Cfz, and it became clear to us that we might be the pioneer to conduct this study for the first time. Positive results with high sensitivity showed the ability of the current validated HPLC method to use in MIC and MBC determination in an accurate and precise method. This method has precedence and superiority over the use of the ordinary and common method for measuring turbidity using spectrophotometry. This is due to the high ability of HPLC to separate the target Cfz peak from all the matrix content, especially at the low concentration levels of Cfz.

HPLC results of the Cfz after the incubation were summarized in Additional file 1: Table S14.

The manifested Cfz assay results confirmed that inhibition of the *B. cepacia* bacterial growth had a direct proportion with the increase in the Cfz conc. Where it was very clear at the lowest Cfz conc, the Cfz peak area and its assay hadn't been detected. On the contrary, by increasing the Cfz conc, the remaining Cfz peak areas were increased and so the assay was increased. This finding confirmed the obtained visual examinations that were manifested in Additional file 1: Fig. S3.

Also, the data that were tabulated in Additional file 1: Table S14 could be used as an index to determine the effective conc of the Cfz to prevent the *B. cepacia* bacterial inhibition to grow as was demonstrated in the determination of the MIC and MBC of Cfz.

### Limitations of the current analysis method

From our point of view, as it became clear to us through the study of MIC and MBC, the composition of the media affects the cefoperazone drug, and therefore we made the negative control. In addition to the possibility of the emergence of new degradation materials in the case of using this method for determination of the mixture components of cefoperazone and sulbactam. So, we will conduct a supplementary study for the current manuscript that studies the stability degradation effect of cefoperazone in the presence of sulbactam at the shelf life.

## Conclusions

The validity of the new analysis method for the quantitation and detection of Cfz at low concentration levels by achieving acceptance criteria was confirmed by the validation guidelines. The use of the current method was verified for the first time in the determination of MIC and MBC especially, for the standard strain of *B. cepacia* ATCC 25416 at MIC equals 250 μg/mL. Whereas the MBC was given at 500 μg/mL. Also, Cfz was determined alone and in combination with sulbactam, which indicates that the current innovative analysis method has high specificity and selectivity. The importance of this method was also evident in the complete separation of Cfz from any degradation products that may be present. The minimum resolution between the Cfz principal peak and the most adjacent related impurity peak is 1.84.

## Supplementary Information

**Additional file 1: Figure S1a.** Cfz chromatogram at a flow rate 0.9 mL/min, Column—1, Buffer 80% & Acetonitrile 20%. **Figure S1b.** Cfz chromatogram at a flow rate 1.1 mL/min, Column—1, Buffer 80% & Acetonitrile 20%. **Figure S1c.** Cfz chromatogram at a flow rate 1.0 mL/min, Column—1, Buffer 82.5% & Acetonitrile 17.5%. **Figure S1d.** Cfz chromatogram at a flow rate 1.0 mL/min, Column—1, Buffer 77.5% & Acetonitrile 22.5%. **Figure S1e.** Cfz chromatogram at a flow rate 1.0 mL/min, Column—1, Buffer 80% & Acetonitrile 20%, Day—2. **Figure S1f.** Cfz chromatogram at a flow rate 1.0 mL/min, Column—2, Buffer 80% & Acetonitrile 20%, Day—2. **Figure S1g.** Cfz chromatogram at a flow rate 1.0 mL/min, Column—3, Buffer 80% & Acetonitrile 20%, Day—2. **Figure S2a.** Forced degradation using acid hydrolysis 0.1 M HCl for 30 min. **Figure S2b.** Forced degradation using base hydrolysis 0.1 M NaOH for 30 min. **Figure S2c.** Forced degradation using H_2_O_2_ 3% w/v hydrolysis for 30 min. **Figure S2d.** Light-forced degradation after 6 h. **Figure S3.** Visual examination of the *B. cepacia bacterial growth* after 24 h. **Table S1.** Repeatability and precision. **Table S2.** Change in the flow rate results (0.9 mL/min–1.1 mL/min). **Table S3.** Change in organic ratio results (17.5–22.5%). **Table S4.** Day-to-day precision results. **Table S5.** Column-to-Column precision results. **Table S6.** Mobile phase composition system suitability. **Table S7.** Resolution factor at different forced degradation states. **Table S8.** Cfz assay for the different local finished products*.* **Table S9.** Cfz after constitution stability study using WFI. **Table S10.** Cfz after dilution stability study using Sodium chloride 0.9%. **Table S11.** Cfz after dilution stability study using Glucose 5%. **Table S12.** Cfz after dilution stability study using Glucose 10%. **Table S13.** Cfz after dilution stability study using Ringer. **Table S14.** Cfz assay after incubation with *B.* *cepacia* of at 37 °C for 24 h.

## Data Availability

All data generated or analyzed during this study are included in this article and the raw data is available from the corresponding author if requested.

## Abbreviations

ACN: Acetonitrile
ATCC: American Type Culture Collection
*B. cepacia*: *Burkholderia cepacia*
BP: British-Pharmacopoeia
Cfz: Cefoperazone
Conc: Concentration
HPLC: High-performance liquid chromatography
UV: Ultraviolet
LOD: Limit of detection
LOQ: Limit of quantitation
MIC: Minimum inhibitory concentration
MBC: Minimum bacteriostatic concentration
MeOH: Methanol
P. As: Peak areas
RSD: Relative standard deviation
STDEV: Standard deviation
USP: United States Pharmacopeia
WFI: Water for injection
