# Supplementary material for: Cefoperazone rapidly and sensitive quantitative assessment via a validated RP-HPLC method for different dosage forms, in-use stability, and antimicrobial activities
Source: BMC Chem. 2023 Jul 12;17(1):72. doi: 10.1186/s13065-023-00989-0 (PMC10337204; doi:10.1186/s13065-023-00989-0)
Supplement: Supplementary file 1 — Additional file 1: Figure S1a. Cfz chromatogram at a flow rate 0.9 mL/min, Column—1, Buffer 80% & Acetonitrile 20%. Figure S1b. Cfz chromatogram at a flow rate 1.1 mL/min, Column—1, Buffer 80% & Acetonitrile 20%. Figure S1c. Cfz chromatogram at a flow rate 1.0 mL/min, Column—1, Buffer 82.5% & Acetonitrile 17.5%. Figure S1d. Cfz chromatogram at a flow rate 1.0 mL/min, Column—1, Buffer 77.5% & Acetonitrile 22.5%. Figure S1e. Cfz chromatogram at a flow rate 1.0 mL/min, Column—1, Buffer 80% & Acetonitrile 20%, Day—2. Figure S1f. Cfz chromatogram at a flow rate 1.0 mL/min, Column—2, Buffer 80% & Acetonitrile 20%, Day—2. Figure S1g. Cfz chromatogram at a flow rate 1.0 mL/min, Column—3, Buffer 80% & Acetonitrile 20%, Day—2. Figure S2a. Forced degradation using acid hydrolysis 0.1 M HCl for 30 min. Figure S2b. Forced degradation using base hydrolysis 0.1 M NaOH for 30 min. Figure S2c. Forced degradation using H2O2 3% w/v hydrolysis for 30 min. Figure S2d. Light-forced degradation after 6 h. Figure S3. Visual examination of the B. cepacia bacterial growth after 24 h. Table S1. Repeatability and precision. Table S2. Change in the flow rate results (0.9 mL/min–1.1 mL/min). Table S3. Change in organic ratio results (17.5–22.5%). Table S4. Day-to-day precision results. Table S5. Column-to-Column precision results. Table S6. Mobile phase composition system suitability. Table S7. Resolution factor at different forced degradation states. Table S8. Cfz assay for the different local finished products. Table S9. Cfz after constitution stability study using WFI. Table S10. Cfz after dilution stability study using Sodium chloride 0.9%. Table S11. Cfz after dilution stability study using Glucose 5%. Table S12. Cfz after dilution stability study using Glucose 10%. Table S13. Cfz after dilution stability study using Ringer. Table S14. Cfz assay after incubation with B. cepacia of at 37 °C for 24 h. [file 13065_2023_989_MOESM1_ESM.docx]

**
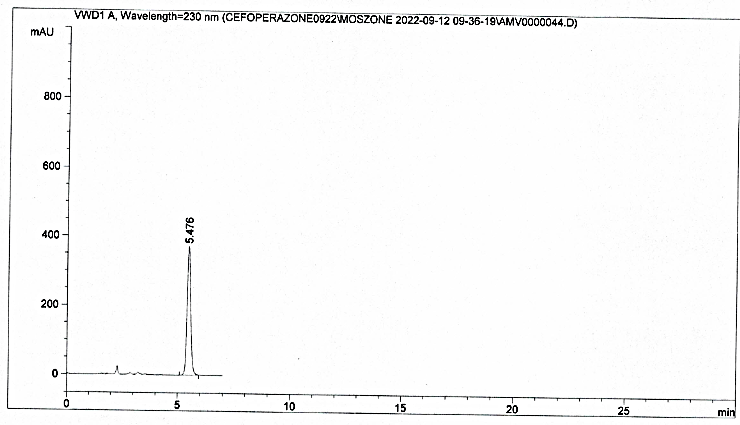

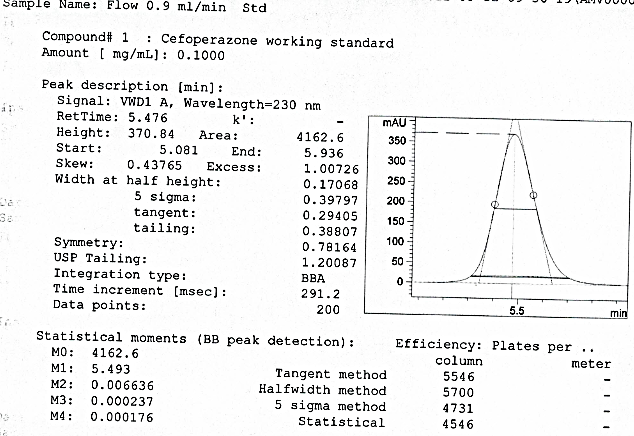
**

**Figure S1a. Cfz chromatogram at a flow rate 0.9mL/min, Column—1, Buffer 80% & Acetonitrile 20%.**

**
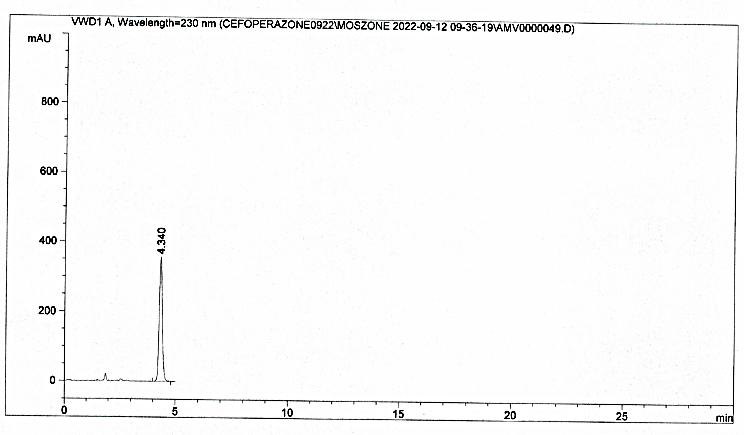

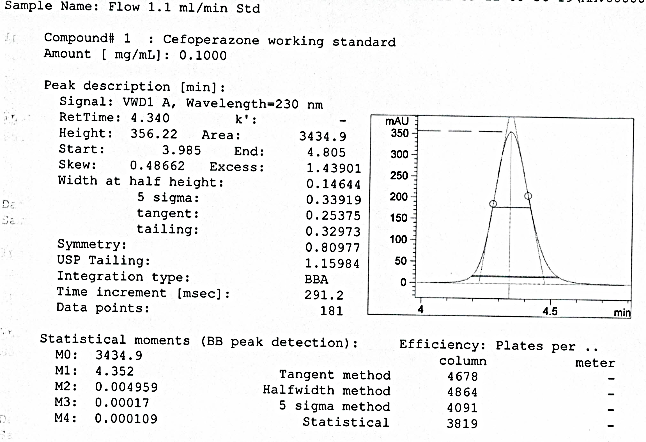
**

**Figure S1b.** **Cfz chromatogram at a flow rate 1.1mL/min, Column—1, Buffer 80% & Acetonitrile 20%.**


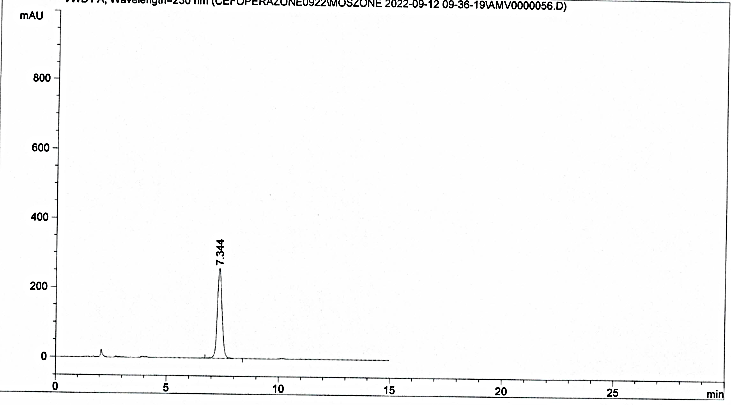

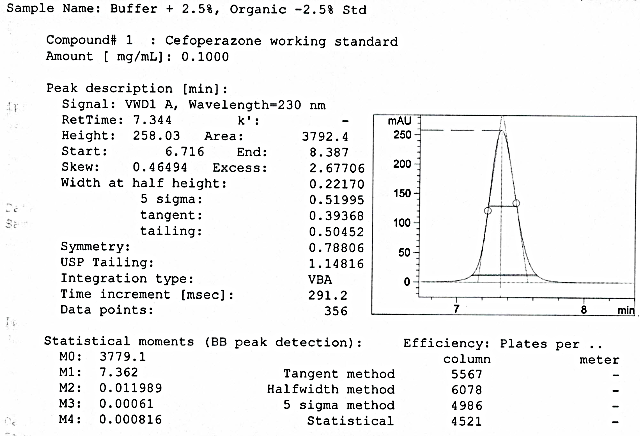


**Figure S1c.** **Cfz chromatogram at a flow rate 1.0mL/min, Column—1, Buffer 82.5% & Acetonitrile 17.5%.**


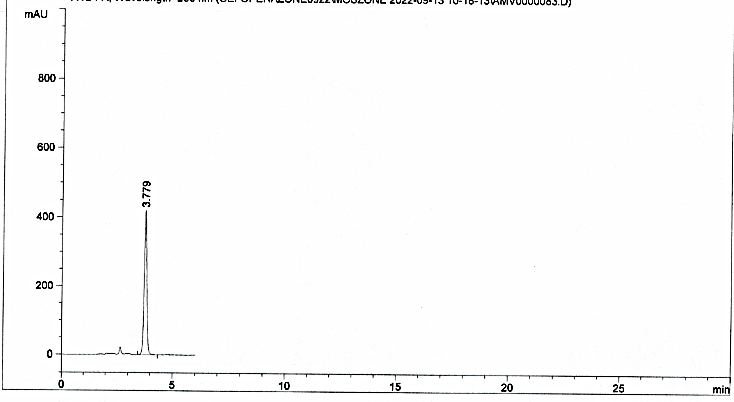

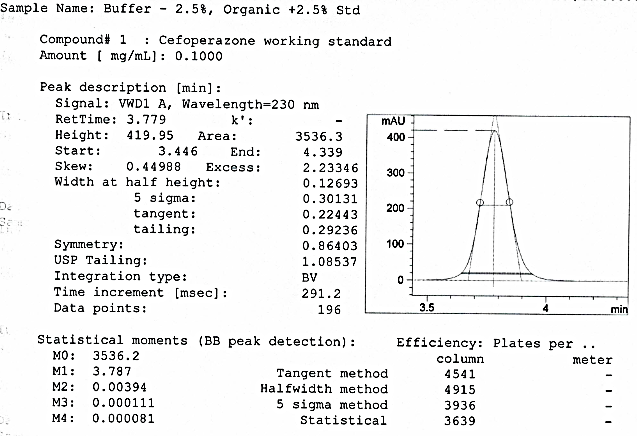


**Figure S1d.** **Cfz chromatogram at a flow rate 1.0mL/min, Column—1, Buffer 77.5% & Acetonitrile 22.5%.**

**
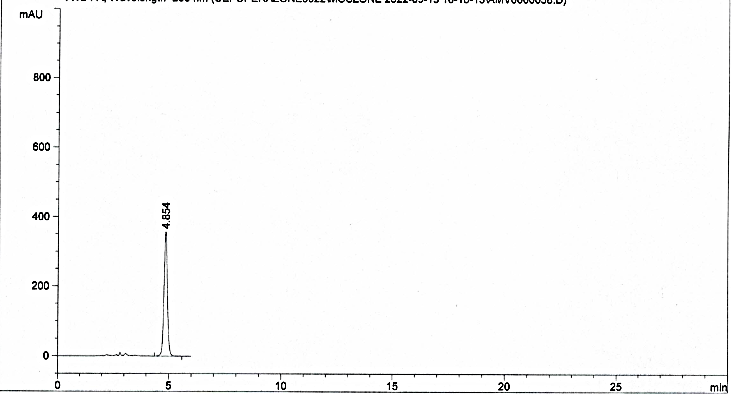

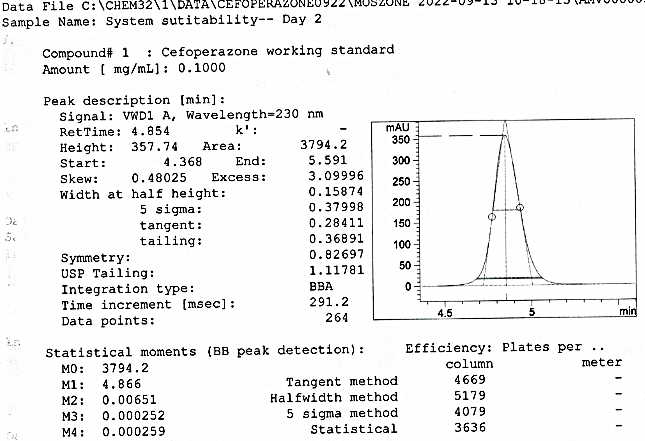
**

**Figure S1e.** **Cfz chromatogram at a flow rate 1.0mL/min, Column—1, Buffer 80% & Acetonitrile 20%, Day---2.**

**
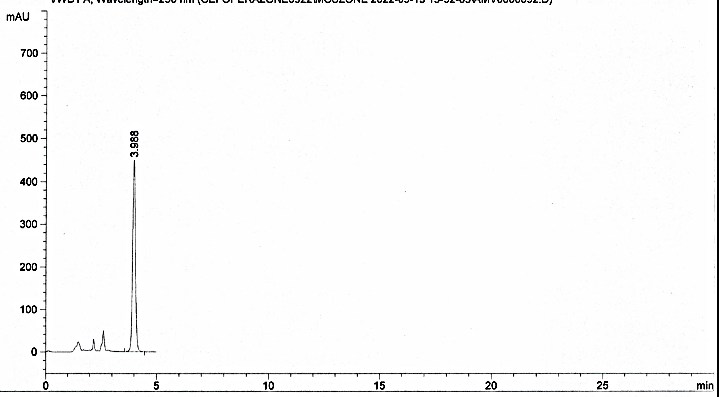

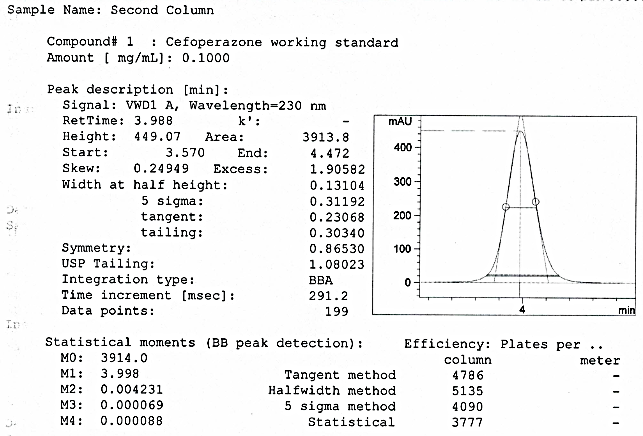
**

**Figure S1f. Cfz chromatogram at a flow rate 1.0mL/min, Column—2, Buffer 80% & Acetonitrile 20%, Day---2.**


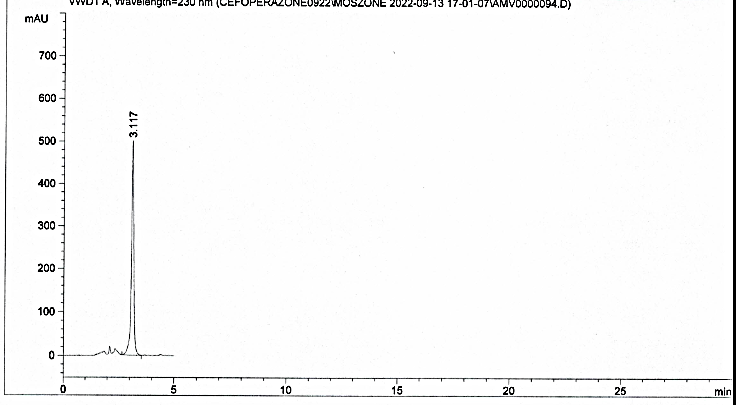

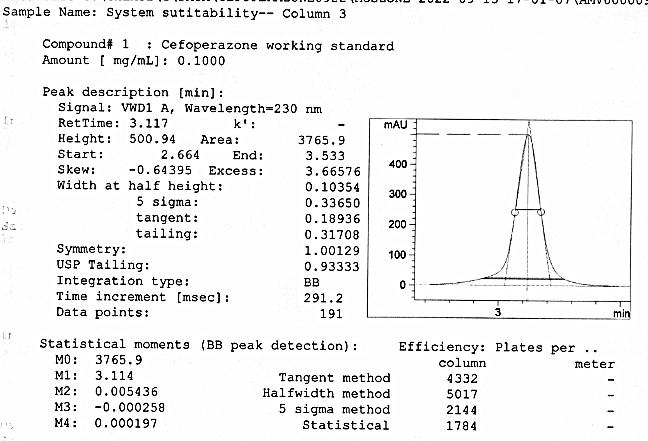


**Figure S1g.** **Cfz chromatogram at a flow rate 1.0mL/min, Column—3, Buffer 80% & Acetonitrile 20%, Day---2.**


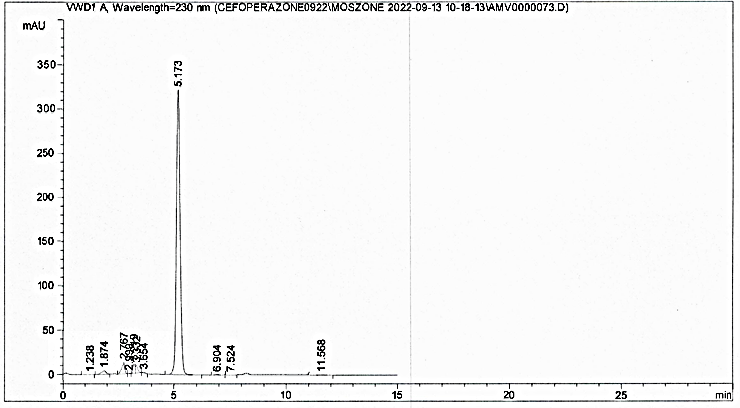


**Figure S2a. Forced degradation using acid hydrolysis 0.1M HCl for 30 minutes.**

**
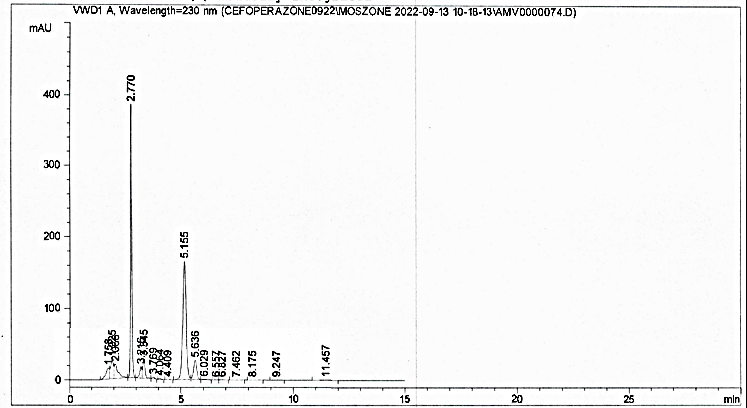
**

**Figure S2b. Forced degradation using base hydrolysis 0.1M NaOH for 30 minutes.**


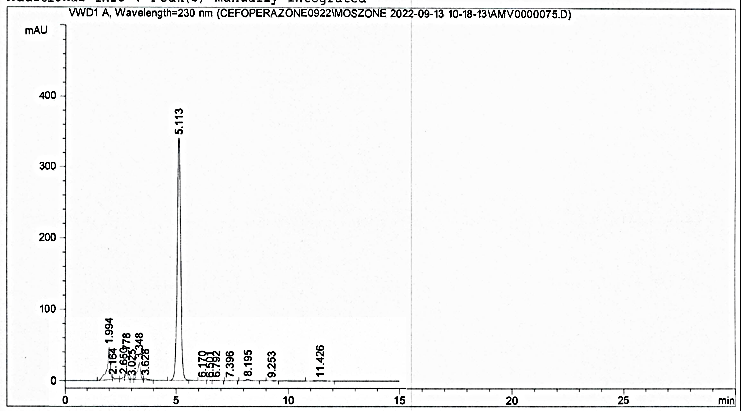


**Figure S2c. Forced degradation using H_2_O_2_ 3% w/v hydrolysis for 30 minutes.**


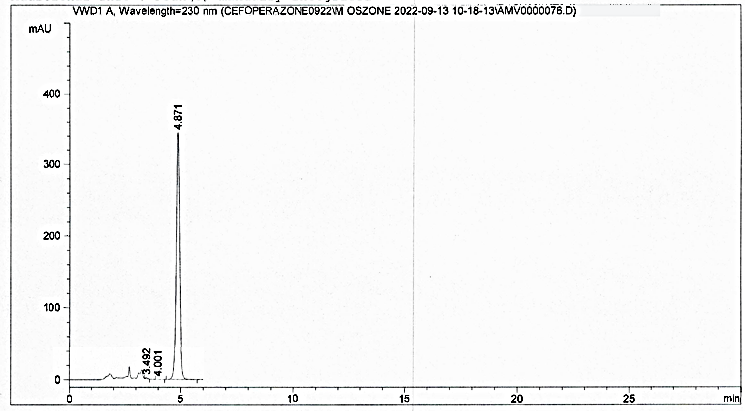


**Figure S2d. Light-forced degradation after 6 hours.**


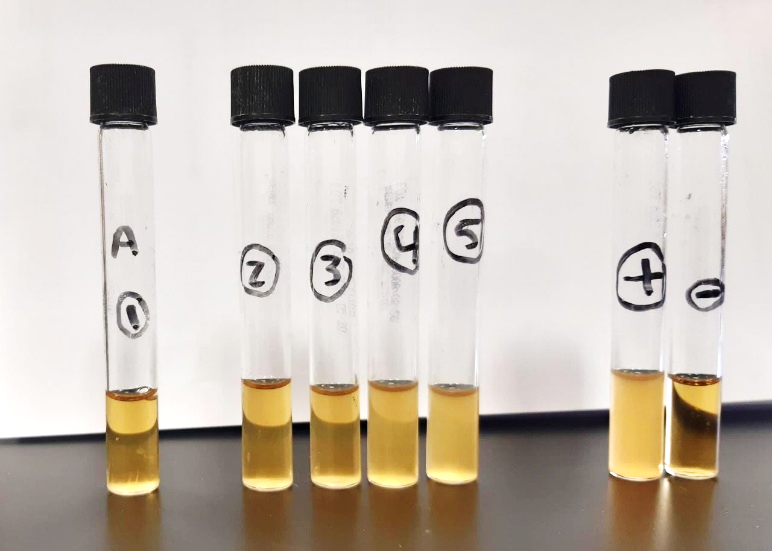


**Figure S3. Visual examination of the *B. cepacia bacterial growth* after 24 hours.**

**Table S1: Repeatability and precision**

| Standard replicate | Day---1 | Day---2 |  | | | |
| --- | --- | --- | --- | --- | --- | --- |
| Weight (g)/ 100mL | 0.01094 g | 0.01095 g |  |  |  |  |
| 1 | 3744.7 | 3794.2 |  |  |  |  |
| 2 | 3746.1 | 3782.9 |  |  |  |  |
| 3 | 3730.9 | 3846.9 |  |  |  |  |
| 4 | 3785.2 | 3794.1 |  |  |  |  |
| 5 | 3791.3 | 3759.1 |  |  |  |  |
| 6 | 3753.8 | 3737.3 |  |  |  |  |
| Mean peak area | 3758.7 | 3785.8 |  |  |  |  |
| STDV | 24.2 | 37.3 |  |  |  |  |
| RSD | 0.64% | 0.98% |  |  |  |  |
| Tailing | 1.15979 | 1.11781 |  |  |  |  |
| Plates | 5411 | 5179 |  |  |  |  |
| Test weight | Day---1  Weight= 0.01094 g/100mL | | | Day---2  Weight= 0.01095 g/100mL | | |
| Determination | Peak area | Actual wt (g) | Actual Assay | Peak area | Actual wt (g) | Actual Assay |
| 1 | 3817.3 | 0.01094 | 101.6 | 3707.6 | 0.01078 | 99.5 |
| 2 | 3816.1 | 0.01098 | 101.2 | 3718.4 | 0.01074 | 100.1 |
| 3 | 3769.7 | 0.01091 | 100.6 | 3726.6 | 0.01081 | 99.7 |
| 4 | 3817.7 | 0.01098 | 101.2 | 3747.3 | 0.01089 | 99.5 |
| 5 | 3771.3 | 0.01089 | 100.8 | 3879.1 | 0.01119 | 100.3 |
| 6 | 3938.7 | 0.01129 | 101.5 | 3720.9 | 0.01081 | 99.6 |
| Mean Assay | 101.1% | | | 99.8% | | |
| STDV | 0.40 | | | 0.34 | | |
| RSD | 0.39% | | | 0.34% | | |
| Pooled P.A | 100.5% | | | | | |
| Pooled STDEV | 0.92 | | | | | |
| Pooled RSD | 0.92% | | | | | |

**Table S2: Change in the flow rate results (0.9mL/min-1.1 mL/min)**

| Standard replicate | Flow(1mL) | Flow(0.9mL) | Flow(1.1mL) |  |
| --- | --- | --- | --- | --- |
| Weight (g) | 0.01094 | 0.01094 | 0.01094 |  |
| 1 | 3744.7 | 4138.2 | 3443 |  |
| 2 | 3746.1 | 4115.3 | 3441.3 |  |
| 3 | 3730.9 | 4164.9 | 3434.5 |  |
| 4 | 3785.2 | 4155.2 | 3444.2 |  |
| 5 | 3791.3 | 4128.8 | 3435.8 |  |
| 6 | 3753.8 | 4133 | 3429.5 |  |
| Mean P.A | 3758.7 | 4139.2 | 3438.1 |  |
| Tests | | | | |
| Weight (g) | 0.01048 | 0.01081 | 0.01081 |  |
| 1 | 3613.1 | 4138.2 | 3443 |  |
| 2 | 3617.3 | 4115.3 | 3441.3 |  |
| 3 | 3612.9 | 4164.9 | 3434.5 |  |
| Mean P.A | 3614.4 | 4139.5 | 3439.6 |  |
| Assay | 100.4% | 101.2% | 101.3% |  |
| Pooled assay | 101.0% | | |  |
| Pooled STDEV | 0.49 | | |  |
| Pooled RSD | 0.49% | | |  |

**Table S3: Change in organic ratio results (17.5%-22.5%)**

| Standard replicate | 20% | 17.5% | 22.5% |  |
| --- | --- | --- | --- | --- |
| Weight (g)/100mL | 0.01094 g | 0.01092 g | 0.01094 g |  |
| 1 | 3744.7 | 3779.1 | 3517.1 |  |
| 2 | 3746.1 | 3756.2 | 3486 |  |
| 3 | 3730.9 | 3784 | 3513.5 |  |
| 4 | 3785.2 | 3784.5 | 3515.4 |  |
| 5 | 3791.3 | 3754.8 | 3554.2 |  |
| 6 | 3753.8 | 3754.7 | 3524.5 |  |
| Mean P.A | 3758.7 | 3768.9 | 3518.5 |  |
| Tests | | | | |
| Weight (g)/100mL | 0.01048 g | 0.01092 g | 0.01092 g |  |
| 1 | 3613.1 | 3779.1 | 3517.1 |  |
| 2 | 3617.3 | 3756.2 | 3486 |  |
| 3 | 3612.9 | 3784 | 3513.5 |  |
| Mean P.A | 3614.4 | 3773.1 | 3505.5 |  |
| Assay | 100.4% | 100.1% | 99.8% |  |
| Pooled assay | 100.1% | | |  |
| Pooled STDEV | 0.30 | | |  |
| Pooled RSD | 0.30% | | |  |

**Table S4: Day-to-day precision results**

| Standard replicate | Day---1 | Day---2 |  |
| --- | --- | --- | --- |
| Weight (g)/100mL | 0.01094 g | 0.01095 g |  |
| 1 | 3744.7 | 3794.2 |  |
| 2 | 3746.1 | 3782.9 |  |
| 3 | 3730.9 | 3846.9 |  |
| 4 | 3785.2 | 3794.1 |  |
| 5 | 3791.3 | 3759.1 |  |
| 6 | 3753.8 | 3737.3 |  |
| Mean P.A | 3758.7 | 3785.8 |  |
| Tests | | | |
| Weight (g)/100mL | 0.01048 g | 0.01091 g |  |
| 1 | 3613.1 | 3728 |  |
| 2 | 3617.3 | 3750.2 |  |
| 3 | 3612.9 | 3732 |  |
| Mean P.A | 3614.4 | 3736.7 |  |
| Assay | 100.4% | 99.1% |  |
| Pooled assay | 99.8% | |  |
| Pooled STDEV | 0.92 | |  |
| Pooled RSD | 0.92% | |  |

**Table S5: Column-to-Column precision results**

| Standard replicate | Column--1 | Column--2 | Column--3 |  |
| --- | --- | --- | --- | --- |
| Weight (g)/100mL | 0.01094 g | 0.01097 g | 0.01091 g |  |
| 1 | 3744.7 | 3864.8 | 3765.9 |  |
| 2 | 3746.1 | 3896.9 | 3781.2 |  |
| 3 | 3730.9 | 3908 | 3806.3 |  |
| 4 | 3785.2 | 3905.9 | 3799.9 |  |
| 5 | 3791.3 | 3887.8 | 3796.6 |  |
| 6 | 3753.8 | 3904.3 | 3859.8 |  |
| Mean P.A | 3758.7 | 3894.6 | 3801.6 |  |
| Tests | | | | |
| Weight (g)/100mL | 0.01048 g | 0.01095 g | 0.01089 g |  |
| 1 | 3613.1 | 3862.2 | 3819 |  |
| 2 | 3617.3 | 3913.8 | 3836 |  |
| 3 | 3612.9 | 3902.9 | 3817.2 |  |
| Mean P.A | 3614.4 | 3893.0 | 3824.1 |  |
| Assay | 100.4% | 100.1% | 100.8% |  |
| Pooled assay | 100.4% | | |  |
| Pooled STDEV | 0.35 | | |  |
| Pooled RSD | 0.35% | | |  |

**Table S6: Mobile phase composition system suitability**

| Replicate | Retention time | USP Tailing | Theoretical Plates |
| --- | --- | --- | --- |
| Day—1 @ 14-09-2022 | 4.906 | 1.13 | 2857 |
| Day—7 @ 21-09-2022 | 4.915 | 1.13 | 2808 |
| Day—14@ 28-09-2022 | 4.893 | 1.14 | 2723 |

**Table S7: Resolution factor at different forced degradation states**

| **Forced degradation item** | **Degradation%** | **Resolution** |
| --- | --- | --- |
| Acid hydrolysis | 1.7 | 5.38 |
| Base hydrolysis | 53.3 | 1.84 |
| Oxidation hydrolysis | 2.3 | 3.93 |
| Light degradation | 3.4 | 3.04 |

**Table S8: Cfz assay for the different local finished products**

| **Local name** | **Manufacturer** | **Strength** | **Batch No.** | **Assay %** |
| --- | --- | --- | --- | --- |
| Peracef | UP pharma | 1.0g | 220001 | 98.0 |
| Peractam | UP pharma | 1.5g | 220005 | 101.5 |
| Trexotaz | Rameda | 1.5g | 210840 | 95.4 |
| Sulbacef | Advocure | 1.5g | 214686 | 99.7 |

**Table S9: Cfz after constitution stability study using WFI**

| **Item** | **Zero h** | **24 h @ 30±2 °C** | **24 h @ 5±3 °C** |
| --- | --- | --- | --- |
| Injection peak area | 3935.7 | 3783.8 | 3912.2 |
|  | 3934.1 | 3808.3 | 3910.9 |
|  | 3948.7 | 3768.5 | 3919.2 |
| Average peak area | 3939.5 | 3786.9 | 3914.1 |
| Assay (%) | 98.0 | 95.5 | 97.3 |

**Table S10: Cfz after dilution stability study using Sodium chloride 0.9%**

| **Item** | **Zero h** | **24 h @ 30±2 °C** | **24 h @ 5±3 °C** |
| --- | --- | --- | --- |
| Injection peak area | 4090.3 | 4058.3 | 3756.6 |
|  | 4057.1 | 4073.2 | 3761.1 |
|  | 4066.5 | 4074.3 | 3750.9 |
| Average peak area | 4071.3 | 4068.6 | 3756.2 |
| Assay (%) | 98.7 | 97.7 | 95.9 |

**Table S11: Cfz after dilution stability study using Glucose 5%**

| **Item** | **Zero h** | **24 h @ 30±2 °C** | **24 h @ 5±3 °C** |
| --- | --- | --- | --- |
| Injection peak area | 4120.9 | 4088.9 | 3642.5 |
|  | 4038.4 | 4110.9 | 3645.3 |
|  | 4018.2 | 4070.6 | 3647.3 |
| Average peak area | 4059.2 | 4090.1 | 3645.0 |
| Assay (%) | 98.1 | 97.4 | 97.9 |

**Table S12: Cfz after dilution stability study using Glucose 10%**

| **Item** | **Zero h** | **24 h @ 30±2 °C** | **24 h @ 5±3 °C** |
| --- | --- | --- | --- |
| Injection peak area | 3963.7 | 3594.5 | 3684.3 |
|  | 3943.0 | 3589.3 | 3676.1 |
|  | 3946.5 | 3604.3 | 3682.1 |
| Average peak area | 3951.1 | 3596.0 | 3680.8 |
| Assay (%) | 98.5 | 97.2 | 96.7 |

**Table S13: Cfz after dilution stability study using Ringer**

| **Item** | **Zero h** | **24 h @ 30±2 °C** | **24 h @ 5±3 °C** |
| --- | --- | --- | --- |
| Injection peak area | 3869.2 | 3836.9 | 3590.3 |
|  | 3844.4 | 3834.2 | 3591.1 |
|  | 3845.9 | 3859.6 | 3579.9 |
| Average peak area | 3853.2 | 3843.6 | 3587.1 |
| Assay (%) | 98.3 | 95.9 | 96.3 |

**Table S14: Cfz assay after incubation with *B.* cepacia of at 37 °C for 24 h**

| **Item** | **Founded peak area** | **Degradation%** | **Cfz (%)** |
| --- | --- | --- | --- |
| Cfz reference working standard in WFI | 3955.4 | ---- | 95.5 |
| Negative control at 1000 µg/mL conc. in Media | 2300.8 | 43.4 | 56.7 |
| Cfz 62.5 µg/mL conc. in media | Not detected | 100 | ---- |
| Cfz 125 µg/mL conc. in media | 4.2 | 99.2 | 0.83 |
| Cfz 250 µg/mL conc. in media | 112.9 | 88.9 | 11.1 |
| Cfz 500 µg/mL conc. in media | 539.7 | 73.4 | 26.6 |
| Cfz 1000 µg/mL conc. in media | 1252.1 | 69.1 | 30.9 |
